# Advancements in understanding the molecular and immune mechanisms of *Bartonella* pathogenicity

**DOI:** 10.3389/fmicb.2023.1196700

**Published:** 2023-06-09

**Authors:** Xiaoxia Jin, Yuze Gou, Yuxian Xin, Jingwei Li, Jingrong Sun, Tingting Li, Jie Feng

**Affiliations:** ^1^Gansu Provincial Key Laboratory of Evidence Based Medicine and Clinical Translation and Lanzhou Center for Tuberculosis Research, School of Basic Medical Sciences, Lanzhou University, Lanzhou, China; ^2^Key Laboratory of Preclinical Study for New Drugs of Gansu Province, School of Basic Medical Sciences, Lanzhou, China; ^3^State Key Laboratory of Veterinary Etiological Biology, College of Veterinary Medicine, Lanzhou University, Lanzhou, China

**Keywords:** *Bartonella*, blood-sucking arthropods, endothelial cells, erythrocytes, antibody, immune escape

## Abstract

Bartonellae are considered to be emerging opportunistic pathogens. The bacteria are transmitted by blood-sucking arthropods, and their hosts are a wide range of mammals including humans. After a protective barrier breach in mammals, *Bartonella* colonizes endothelial cells (ECs), enters the bloodstream, and infects erythrocytes. Current research primarily focuses on investigating the interaction between *Bartonella* and ECs and erythrocytes, with recent attention also paid to immune-related aspects. Various molecules related to *Bartonella*’s pathogenicity have been identified. The present review aims to provide a comprehensive overview of the newly described molecular and immune responses associated with *Bartonella*’s pathogenicity.

## Introduction

Bartonellae are Gram-negative facultative intracellular bacteria (Brenner et al., 1993) that were first described in 1909. Since the last reclassification in 1993, the number of Bartonellae species has increased to 45 as of Okaro et al. (2017), and new species continue to be identified in recent years (Gutierrez et al., 2020; do Amaral et al., 2022a,b; Liu et al., 2022). Phylogenetic analyses have categorized the genus *Bartonella* into three phylogenetic clades, which include the honeybee symbiont *Bartonella apis*, the pathogenic *Bartonella tamiae*, and the eubartonellae, which are further separated into *Bartonella australis* and four distinct lineages (Segers et al., 2017). *Bartonella bacilliformis* and *Bartonella ancashensis*, which are human pathogens, belong to lineage 1. *Bartonella* species specific to ruminants are found in lineage 2. The most abundant *Bartonella* species are those in lineage 3 and 4, which infect a diverse range of mammalian hosts (Wagner and Dehio, 2019). Recently, two bat-associated *Bartonella* strains showed significant differences with other lineages in phylogenetic relationship analysis (Goncalves-Oliveira et al., 2023). Transmission of Bartonellae between hosts is primarily mediated by diverse blood-sucking arthropod vectors such as fleas, body lice, ticks, sandflies, and others (Breitschwerdt et al., 2010a). These pathogens have a broad range of mammalian hosts, including, but not limited to, primates, rodents, and cats (Gundi et al., 2004; Dehio, 2005). However, each *Bartonella* species is typically adapted to a specific mammalian host (Deng et al., 2012).

In arthropods, the life cycle of most *Bartonella* species is divided into replication in the midgut of intestinal tracts and spreading through excretion (Foil et al., 1998). The pathogen is shed within arthropod feces onto mammalian skin and can be superficially inoculated into the derma by scratching or biting (Okujava et al., 2014). The derma-niched *Bartonella* might penetrate the endothelial cells (ECs) facilitated by dendritic cells (DCs) and Bartonella effector proteins (Beps) (Siamer and Dehio, 2015; Fromm and Dehio, 2021). Subsequently, the ECs-residing bacteria enter the bloodstream, invade erythrocytes, multiply inside them, and await the next round of transmission when arthropods bite the infected mammalian host again (Harms and Dehio, 2012; Pulliainen and Dehio, 2012; Fromm and Dehio, 2021).

*Bartonella bacilliformis*, *Bartonella quintana*, and *Bartonella henselae* are three major species that trigger pathological angiogenesis during infection in humans. These three species are the etiological agents of Carrion’s disease, trench fever, and cat scratch disease (CSD), respectively (Rolain et al., 2004; Breitschwerdt et al., 2010a; Harms and Dehio, 2012; Liu et al., 2012). The symptoms and syndromes of bartonelloses are diverse, ranging from bacillary angiomatosis (BA), bacillary peliosis hepatis, chronic asymptomatic bacteremia, and infectious endocarditis, to neurological disorders. CSD, a relatively common zoonotic infection acquired from cats or cat fleas carrying *B. henselae*, is characterized by enlarged regional lymph nodes and fever, particularly in children and adolescents (Biancardi and Curi, 2014; Chang et al., 2016; Okaro et al., 2021). Atypical manifestations of CSD, such as hepatic and/or splenic lesions, discitis, granulomatous conjunctivitis, endocarditis, myocarditis, neuroretinitis, osteomyelitis, and encephalomeningitis, can mimic serious disorders. Moreover, *B. henselae* and *B. clarridgeiae* have been detected in blood samples from human donors at a Brazilian blood bank (Pitassi et al., 2015), indicating their presence in healthy humans, and highlighting the possibility of undetected cases of *Bartonella* infection in humans. *B. bacilliformis*, the pathogen of Carrion’s disease, is primarily found in the Andean valleys of South America (Sanchez Clemente et al., 2012). This disease is characterized by an acute phase, known as Oroya fever, marked by fever, pallor, hemolytic anemia, myalgia, and arthralgia, followed by a chronic phase characterized by the development of vascular proliferative lesions on the skin, known as verruga peruana, which can persist for several months or even years (Maguina et al., 2009; Harms and Dehio, 2012; Sanchez Clemente et al., 2012; Gomes et al., 2016a). *B. quintana*, the etiological agent of trench fever, is naturally restricted to human hosts and louse vectors. The pathogen is now frequently identified among urban homeless and marginalized populations in the United States and Europe, and is responsible for various conditions, including endocarditis, pericardial effusion, bacillary angiomatosis-peliosis, and even asymptomatic bacteremia (Leibler et al., 2016; Sasaki et al., 2021).

This review intends to summarize recent findings about the pathogenicity of *Bartonella* and the immune response in hosts elicited by the infection, to provide valuable insights for future research.

## Blood-sucking arthropods as vectors for *Bartonella* transmission

**FIGURE 1:**
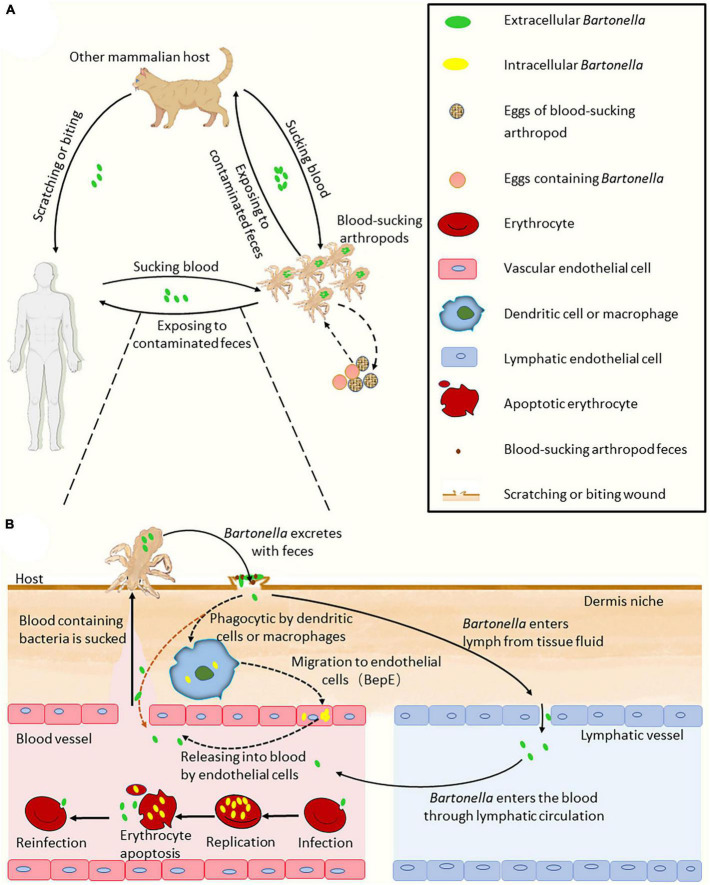
Model of *Bartonella* infection cycle. **(A)** The process of *Bartonella* transmission in the hosts. **(B)** The ways of Bartonellae enter the blood. There are two ways of Bartonellae with VirB/VirD4 T4SS enter the blood. The first way may be through the host’s macrophages, endothelial cells and other migration into the blood. The second way is through lymphatic circulation into the blood. Bartonellae absent of VirB/VirD4 T4SS, such as *B. bacilliformis*, may enter the blood with feces directly through the wound bitten by sand flies (the orange dashed line arrow). In the figure, the solid line arrow indicates the confirmed process, and the dashed line arrow indicates conjecture. Some elements in the figure were drawn by Figdraw.

Blood-sucking arthropods are important for the transmission of *Bartonella*. Various blood-sucking arthropods have been identified as vectors capable of transmitting *Bartonella*, such as the sand flies *Lutzomyia verrucarum* for *B. bacilliformis* (Minnick et al., 2014), cat flea *Ctenocephalides felis* for *B. henselae* (Chomel et al., 2009; Harms and Dehio, 2012; Duscher et al., 2018), human body lice *Pediculus humanus corporis* for *B. quintana* (Byam and Lloyd, 1920), and the flea *Ctenophthalmus nobilis* for *Bartonella grahamii* and *Bartonella taylorii* (Bown et al., 2004). Most Bartonellae reside in the intestinal tract of arthropods, where they replicate and are eventually transmitted to mammalian hosts through vector defecation (Figure 1B; Chomel et al., 2009). Although *Bartonella* bacteria (such as *B. henselae*) typically survive in arthropod feces for several days (Higgins et al., 1996), *B. quintana* remains infectious for up to 1 year in louse feces (Kostrzewski, 1950; Chomel et al., 2009), making it more likely for hosts to contract an infection. Bartonellae can also infect the salivary glands of arthropods, as evidenced by the transmission of *B. henselae* through tick saliva to an artificial membrane feeding system (Cotte et al., 2008). Additionally, the transovarial transmission of *B. henselae* Marseille, *Bartonella schoenbuchensis* DSMZ 13525, and *B. grahamii* ATCC700132 from female tick *Ixodes ricinus* to their offspring via eggs may also occur, as *Bartonella* DNA has been detected in eggs laid by the three kinds of *Bartonella*-positive female ticks and in hatched larvae (Figure 1A; Krol et al., 2021). The spread of *B. bacilliformis* by *L. verrucarum* represents an exception of vector transmission. The current research has shown that *B. bacilliformis* can colonize the midgut of *L. verrucarum* (Battisti et al., 2015), yet the mechanism by which midgut-residing *B. bacilliformis* in sandflies enters human hosts remains unclear (Garcia-Quintanilla et al., 2019).

Climate change indirectly affects the spread of *Bartonella* by perturbing the proliferation of blood-sucking arthropods (Chamberlin et al., 2002). When the number of blood-sucking arthropods increases during warm and humid conditions such as during an El Niño, the infection rate of *Bartonella* also seems to increase correspondingly (Chamberlin et al., 2002; Clemente et al., 2016). These findings highlight the critical role of blood-sucking arthropods in the transmission of *Bartonella*.

The flea *C. felis* is the primary means of transmitting *B. henselae* from cat to cat. Studies have shown that healthy cats do not contract *Bartonella* infection or seroconversion after sharing food or playing with highly bacteremic cats (Chomel et al., 1996; Abbott et al., 1997). However, fleas that have taken a blood meal from highly bacteremic cats can effectively transmit *B. henselae* to specific-pathogen-free (SPF) cats (Figure 1A; Chomel et al., 1996). Although there is no evidence to prove that *B. henselae* can be spread among cats through salivation, *B. henselae* DNA has been detected in the saliva of infected cats (Oskouizadeh et al., 2010). *Bartonella* may be present in the saliva of infected mammals, but the bacteria are fastidious haemophilia (Liu and Biville, 2013), and therefore, the concentration of *Bartonella* in the blood of a highly bacteremic host is likely higher than that in its saliva. Furthermore, different mechanisms exist in certain arthropods that enable the transmission of *Bartonella* from arthropods to hosts. It is important to note that the transmission of *Bartonella* from cat to human is usually achieved through cat scratches due to contact with *B. henselae-*contaminated flea feces.

The importance of some arthropod vectors for the transmission of *B. henselae* underscores the likelihood that this applies to most *Bartonella* species (Pulliainen and Dehio, 2012). After *Bartonella*-contaminated feces are inoculated to the wound of the host, the bacteria invade the dermis layer of the host and begin their journey of invasion (Figure 1B).

## Migration and blood entry of *Bartonella* in mammalian host

*Bartonella* initiates its infection cycle in a mammalian host after replication in the midgut of arthropod vectors (Harms and Dehio, 2012). When these arthropods suck blood from mammals, the affected area on the host’s skin experiences irritation, followed by scratching that can lead to the inoculation of *Bartonella*-containing insect feces into the dermis. Transmission of *Bartonella* can also occur through traumatic contact with infected animals (Chomel et al., 2009). After superficially inoculating the dermis, the bacteria sequentially access the dermal and blood-seeding niches. During dermal niche colonization, migrating cells such as DCs or macrophages are likely kidnapped by *Bartonella* to reach the blood-seeding niche (Fromm and Dehio, 2021). Transmission from the dermal niche to the blood-seeding niche may also occur via the lymphatic system (Hong et al., 2017). In the blood-seeding niche, the cell type is still puzzling and the bacteria most likely colonize ECs (Eicher and Dehio, 2012). It is believed that the blood-seeding niche releases bacteria into the bloodstream periodically, which invade erythrocytes and replicate until the critical limit of eight bacteria on average per erythrocyte is reached (Litwin and Johnson, 2005; Pulliainen and Dehio, 2012). After the apoptosis of infected erythrocytes, bacterial cells are released again into the bloodstream to invade new erythrocytes for replication, which may partly explain the periodic occurrence of host bacteremia after infection (Harms and Dehio, 2012). Once arthropods bite the infected mammals again, *Bartonella* enters the next round of transmission (Figure 1A; Harms and Dehio, 2012; Fromm and Dehio, 2021).

Bartonellae (lineage 3 and lineage 4), along with other bacteria like *Helicobacter pylori*, *Legionella pneumophila*, and *Brucella* spp. (Cascales and Christie, 2003; Harms et al., 2017), utilize the VirB/VirD4 type IV secretion system (T4SS) as a key virulence factor to infect humans and other mammals’ target cells (Cascales and Christie, 2003). The pathogens translocate Bartonella effector proteins (Beps) into host cells via the VirB/VirD4 T4SS, which orchestrates multiple cellular processes in host cells, including modulating the immune response and subverting cellular functions to benefit the bacterial survival and proliferation (Okujava et al., 2014; Sorg et al., 2020). The VirB/VirD4 T4SS and the translocated Beps are the best characterized *Bartonella*-specific virulence factors. The invasion of *B. henselae* into human ECs occurs in two distinct pathways, either as a single bacterium through endocytosis or as bacterial aggregates in the form of invasomes (Truttmann et al., 2011). Induced by F-actin rearrangements and stress fiber formation, the formation of invasome relies on the functional VirB/VirD4 T4SS associated with either BepG alone or the combination of BepC and BepF (Truttmann et al., 2011). BepF is comprised of three Bep intracellular delivery domains, with two non-terminal domains triggering invasome formation in conjunction with BepC (Wagner and Dehio, 2019). BepC contributes to the formation of the invasome by modulating the F-actin cytoskeleton. Recently, it has been discovered that BepC triggers stress fiber formation by activating the RhoA GTPase signaling cascade through the recruitment of the Rho guanine nucleotide exchange factor H1 (GEF-H1) to the plasma membrane (Marlaire and Dehio, 2021).

The VirB/VirD4 T4SS system also plays a crucial role in the migration of *Bartonella* from the dermal niche to the blood-seeding niche. It is achieved by translocating BepE into host cells, which inhibits cell fragmentation caused by BepC or other Beps and ensures the migration of *Bartonella*-infected DCs to deliver the pathogen from the derma site to the blood-seeding niche (Okujava et al., 2014). The distributional pattern of VirB/VirD4 T4SS and Beps is proposed to correlate with arthropod vectors’ blood-feeding behavior and the mode of bacterial transmission (Dehio and Tsolis, 2017). Bartonellae that are absent of VirB/VirD4 T4SS, such as *B. bacilliformis*, are exclusively transmitted by sandflies. However, sandflies display a violent mode of blood-feeding by damaging microvessels of the skin to allow them to access the freshly draining blood (Ribeiro, 1995; Serafim et al., 2021), which potentially provides a direct route for *Bartonella* to invade the bloodstream (Dehio and Tsolis, 2017).

Furthermore, the presence of either *B. henselae* or *B. quintana* enhances the proliferation of human ECs and prevents apoptosis. This anti-apoptotic effect is mediated by the pathogen’s BepA protein, which interacts with human adenylyl cyclase 7 (AC7), followed by elevated cAMP levels (Pulliainen et al., 2012). The prolonged survival of host cells allows for an efficient time course for intracellular bacteria to have multiple replications, which is believed to be critical for a successful infection from the primary niche to the bloodstream. However, the BepA ortholog in *B. tribocorum* lacks anti-apoptotic activity (Dehio, 2008), indicating that orthologous BepA proteins may have varying functions in different bacteria.

## Infection of endothelial cells (ECs)

**FIGURE 2:**
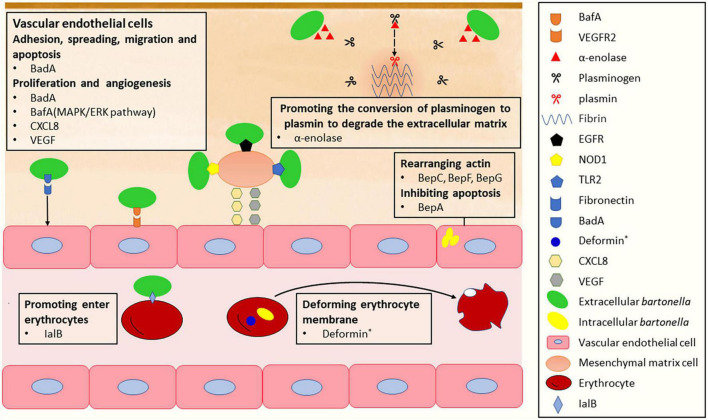
The mechanism of *Bartonella* infects vascular endothelial cells, mesenchymal stromal cells and erythrocytes. *Deformin is only found in *B. bacilliformis* and *B. henselae*.

*Bartonella* species have been found to inhabit various host cells, including mononuclear phagocytes, CD34^+^ progenitor cells, and mesenchymal stromal cells (MSCs) (Mändle et al., 2005), but the vascular endothelium is considered the primary blood-seeding niche for *Bartonella* colonization in the mammalian host (Deng et al., 2012, 2019). To infect the ECs of blood vessels in a mammalian host, Bartonellae must first traverse the extracellular matrix of the cells. The degradation of extracellular matrix proteins has been confirmed to be facilitated by the interaction between fibrinolysis and several pathogen proteins (Lähteenmäki et al., 2001). *Bartonella* utilizes a metabolic enzyme known as α-enolase or phosphopyruvate hydratase, which is involved in the synthesis of pyruvate, to act as a plasminogen receptor and mediate the activation of plasmin and extracellular matrix degradation (Figure 2; Díaz-Ramos et al., 2012; Cappello et al., 2017). Plasmin, the proteolytically active form of plasminogen, is responsible for promoting fibrin dissolution in the extracellular matrix of host cells (Keragala and Medcalf, 2021). The sequence of α-enolase in *B. henselae* is highly homologous to that of many other *Bartonella* species, and they all possess typical plasminogen-binding modes. Accordingly, the α-enolase of *Bartonella* has been hypothesized to function as a plasminogen-binding protein, which has been recently confirmed in experiments (Deng et al., 2019). As a result, the enolase-plasminogen interaction is identified as one potential mechanism exploited by *Bartonella* to loosen and degrade the extracellular matrix of ECs before entering (Deng et al., 2019).

To efficiently adhere to extracellular matrix proteins of ECs, *Bartonella* expresses surface adhesins, such as Bartonella adhesin A (BadA) in *B. henselae* (Riess et al., 2004), variably expressed outer membrane proteins (Vomps) in *B. quintana* (Zhang et al., 2004), and *Bartonella* repeat protein A (BrpA) in *Bartonella vinsonii* (Gilmore et al., 2005). These adhesins belong to the trimeric autotransporter adhesin (TAA) family (Linke et al., 2006). Among them, VompC confers the ability of *B. quintana* to bind collagen IV in extracellular matrix proteins of ECs; BadA is essential for the attachment of *B. henselae* to extracellular matrix proteins including collagen, laminin, and fibronectin, with fibronectin being particularly important (Kaiser et al., 2008; Muller et al., 2011). Fibronectin orchestrates various cellular processes of ECs, including cell adhesion, spreading, migration, proliferation, and apoptosis (Patten and Wang, 2021; Vaca et al., 2022). The binding of BadA to fibronectin occurs at repetitive motifs in the neck/stalk region of BadA and is a cumulative effect leading to rapid saturation (Thibau et al., 2022b). This interaction helps the bacteria to adhere to host cells. As another important virulence factor in *Bartonella*, BadA was described to negatively affect the Beps-translocating activity of VirB/D4 T4SS, while the function of BadA itself remain intact when both factors were co-expressed in *B. henselae* (Lu et al., 2013).

The outer membrane protein BadA is also responsible for *Bartonella*-induced vasoproliferation. It activates hypoxia-inducible factor-1 and stimulates the secretion of pro-angiogenic cytokines, such as vascular endothelial growth factor (VEGF) and C-X-C motif chemokine ligand (CXCL) 8 (Riess et al., 2004; Kempf et al., 2005; McCord et al., 2006), contributing to *Bartonella*-induced vasoproliferation. However, the observed BadA-dependent VEGF secretion was limited to certain cells and not in *B. henselae*-infected ECs (Kempf et al., 2001). Another autotransporter protein, *Bartonella* angiogenic factor A (BafA), is identified as a key *Bartonella*-derived mitogenic factor (Tsukamoto et al., 2020, 2022). BafA acts as a VEGF analog that promotes angiogenesis by binding to vascular endothelial growth factor receptor-2 (VEGFR2) on the EC surface and activating the MAPK/ERK pathway, which facilitates EC proliferation, tube formation, and subsequent angiogenesis (Tsukamoto et al., 2020). BafA family proteins are common among many *Bartonella* species, and BafA-triggered angiogenesis plays a central role in the formation of vasoproliferative lesions during *Bartonella* infection. Studies have shown that *B. quintana*, *B. henselae*, and *B. elizabethae* induce angiogenesis and proliferation by stimulating the VEGFR2 signaling pathway through the production of BafA (Tsukamoto et al., 2020, 2022; Suzuki et al., 2023). In addition, other cell types recruited to the vasoproliferative lesions, including monocytes, macrophages, and MSCs, stimulate EC proliferation through the production of VEGF and CXCL8. In particular, MSCs distributed in various tissues, including the bone marrow and the adipose tissue, play the role of a *B. henselae* reservoir and modulator of EC functions. *B. henselae*-infected MSCs release angiogenic factors, such as CXCL8, VEGF, etc., leading to the induction of a proangiogenic phenotype in ECs. Toll-like receptor 2 (TLR2), nucleotide-binding oligomerization domain-containing protein 1 (NOD1), and epidermal growth factor receptor (EGFR) are identified as the receptors involved in the recognition of *B. henselae* by MSCs (Figure 2; Scutera et al., 2021). Meanwhile, *B. henselae*-infected MSCs also release proinflammatory chemokines, which recruit monocytes/macrophages in the vasoproliferative lesions. The angiogenic factors produced by phagocytic cells play a central role in mediating angiogenesis (Resto-Ruiz et al., 2002; McCord et al., 2006; O’Rourke et al., 2015). The main virulence factors involved in the invasion of the ECs are summarized in Table 1.

**TABLE 1:** The main virulence factors involved in the invasion of endothelial cells and erythrocytes.

Virulence factor	Functions	Lineages	References
Trw type IV secretion system	Adhesion to erythrocyte surface	*Bartonella australis;* Lineage 4	Harms and Dehio, 2012
BadA/Vomps	Binding to extracellular matrix proteins	*B. henselae/B. quintana* (lineage 4)	Riess et al., 2004
Deformin	Damage in erythrocyte membranes	*B. bacilliformis* (lineage 1); *B. henselae* (lineage 4)	Mernaugh and Ihler, 1992; Iwaki-Egawa and Ihler, 1997
VirB/VirD4-T4SS, Bartonella effector proteins	Inhibition of apoptosis, proinflammatory activation, modulation of angiogenesis, invasome formation	*B. ancashensis* (lineage 1); Lineage 3–4	Wagner and Dehio, 2019
BafA	Angiogenesis	*B. quintana*, *B. henselae* and *B. elizabethae* (lineage 4)	Tsukamoto et al., 2020, 2022
CFA	Invasion of erythrocytes	Lineage 1–4	Siewert et al., 2022b
IalB protein	Promoting enter erythrocytes	*B. bacilliformis* (lineage 1); *B. henselae* (lineage 4)	Mitchell and Minnick, 1995; Coleman and Minnick, 2001

## Infection of erythrocytes

The induction of intra-erythrocytic bacteremia is a hallmark of Bartonellae infection in mammalian hosts. The infection process of erythrocytes by *Bartonella* is divided into three main stages: adhesion, erythrocyte deformation, and invasion, with various virulence factors involved (Table 1).

The initial step is the adhesion of *Bartonella* to erythrocytes. In some *Bartonella* species, a type of T4SS called Trw has been identified as a key factor promoting host-specific erythrocyte infection, leading to prolonged bacteremia (Frank et al., 2005). Upon attachment, Bartonellae invade and penetrate mature erythrocytes, although the exact mechanism of this process is not fully understood. However, several necessary factors have been identified. The invasion-associated locus B genes (*ialB*) are crucial for the invasive behavior of the bacteria. IalB protein locates on the outer membrane of *B. henselae* (Coleman and Minnick, 2001; Chenoweth et al., 2004), and the inner membrane of *B. bacilliformis* (Mitchell and Minnick, 1995). The loss of *ialB* does not significantly affect bacterial adhesion, yet it leads to a ten-fold reduction in the number of bacteria residing in erythrocytes (Figure 2; Deng et al., 2016). Another important factor is deformin, a small molecule with protease resistance, heat resistance, and high affinity with albumin (Iwaki-Egawa and Ihler, 1997; Hendrix and Kiss, 2003). During infection, *B. bacilliformis* interacts with human erythrocytes to produce trenches, pits, conical invaginations, and internal vacuoles in the erythrocyte membrane (Mernaugh and Ihler, 1992; Xu et al., 1995), which is stimulated by deformin (Figure 2). Similarly, deformation activity on the erythrocyte membrane has also been reported in *B. henselae* (Iwaki-Egawa and Ihler, 1997).

A recent study showed that the CAMP-like factor autotransporter (CFA) of *B. taylorii* is crucial for infecting erythrocytes. Mice infected with a mutant strain lacking the *cfa* locus remained free of bacteremia, while the wild-type strain caused infection (Siewert et al., 2022b). CFA was first identified as an autotransporter virulence protein with potential cohemolysin activity in *B. henselae* (Litwin and Johnson, 2005). Autotransporters are a family of proteins secreted by gram-negative bacteria through the type V secretion mechanism, and they transport themselves through the outer membrane. The extracellular passenger domain at the N-terminal region of autotransporters often binds to a β-barrel folded by the C-terminal region in the bacterial outer membrane (Tame, 2011). Comparative genomics analysis has shown that the *cfa* locus is present in all Eubartonellae, with a hypervariable antigenic region (Siewert et al., 2022b). Meanwhile, CFA is one of the major targets for protective neutralizing antibodies that can prevent the attachment of pathogens to erythrocytes independent of complement or Fc receptors in hosts (Siewert et al., 2022b).

The undetectable situation of *Bartonella* after erythrocyte invasion plays a significant role in its spread, aided by the mobility and the lack of organelles in mature red blood cells, posing challenges for treatment selection. Research on *Bartonella* will provide insight into intracellular parasitology.

## Host immune response after *Bartonella* invasion

*Bartonella* can cause a broad spectrum of diseases in humans, and the severity of symptoms is closely related to the immune status of the patients. It can be self-limited in immunocompetent individuals (Windsor, 2001; Gai et al., 2015), and it also can be very serious and even fatal for individuals with human immunodeficiency virus (HIV) or advanced immunosuppression (Mosepele et al., 2012). Bartonellae can establish long-lasting intraerythrocytic bacteremia, therefore, the intraerythrocytic cellular niche in host cells poses a daunting challenge to antibacterial immune defense. The lack of the major histocompatibility complex (MHC) on the cell surface of erythrocytes prevents antigen presentation, making MHC-dependent cytotoxicity an ineffective response. However, other immune responses are employed by the host to eliminate the pathogen.

Macrophages and DCs are innate immune cells that are vital for host’s defense against *Bartonella* invasion. *In vitro*, co-incubation of murine macrophage cell line J774 with *B. henselae* led to rapid internalization of the bacterium by the macrophages. The phagocytosis of unstimulated murine macrophages to *B. henselae* achieved full saturation within 4 h, accompanied by a significant increase in the expression of tumor necrosis factor α, interleukin (IL)-1β, and IL-6 by J774 (Musso et al., 2001). Following phagocytosis of *B. henselae*, DCs highly express the aforementioned three cytokines and upregulate the expression of CXCL8, CXCL1, and CXCL13, which recruit neutrophils and B cells to the site of infection (Vermi et al., 2006). The secretion of pro-inflammatory cytokines contributes to the formation of characteristic granuloma in CSD, which promotes the confinement of *B. henselae* to specific sites, limiting its spread within the host.

**FIGURE 3:**
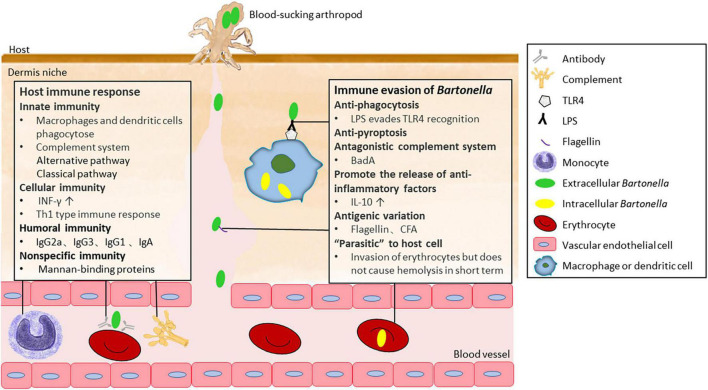
The immune response of the host to *Bartonella* and immune escape mechanism of *Bartonella*.

Mounting antibody responses that neutralize the pathogen is proved to be critical for clearing *Bartonella* cells in the blood of infected mice (Koesling et al., 2001). Studies have demonstrated that antibodies can prevent bacterial attachment to erythrocytes and suppress bacteremia independent of complement or Fc receptors (Pulliainen et al., 2012). The antibodies that interfere with *B. taylorii* adhesion to erythrocytes *in vitro* belong to the IgG2a and IgG3 isotypes (Siewert et al., 2022b; Figure 3). In this humoral immune defense process, the bacterial surface determinant CFA was recently identified as a target for protective antibodies (Siewert et al., 2022b). Furthermore, antibodies against *B. bacilliformis* could confer long-term immune protection. Individuals living in high-risk areas for *B. bacilliformis* have been found to possess significantly higher serum IgG levels specific to *B. bacilliformis* compared to those living in areas where the first outbreak of Carrion’s disease occurred (Gomes et al., 2016b). During CSD infection, IgG and IgA are the main antibodies induced, with IgG1 being the major subclass of IgG (McGill et al., 1998). Specific IgM is the predominant antibody during the acute infectious phase in *B. bacilliformis*-infected patients and elevated specific IgG levels indicate a history of infection (Pons et al., 2017). However, *in vitro* experiments have shown extensive cross-reactivity between *B. henselae* to various microorganisms, such as *Treponema pallidum* and *Chlamydia* group (McGill et al., 1998). The above humoral immune processes can be seen in Figure 3.

The complement system, an important part of the innate immune system, plays a key role in defending against *Bartonella* before it enters erythrocytes in blood vessels and other cell types in peripheral tissues. An *in vitro* experiment showed that the alternative pathway of the complement system was primarily involved, with activation of the classical pathway also detected when human-derived non-immune serum was exposed to *B. henselae* (Rodriguez-Barradas et al., 1995; Figure 3).

In addition, humoral immune defense is essential in preventing and eliminating vertical transmission of *Bartonella*. Vertical transmission of *Bartonella* exists in mice and has been reported in a patient (Breitschwerdt et al., 2010b). Siewert et al. (2022a) recently found that only B cell-deficient offspring developed persistent bacteremia upon vertical transmission of *B. taylorii* in mice, whereas the corresponding wild-type offspring cleared the infection and developed protective immune memory. This result severely challenges the proposal that immunological tolerance in offspring due to vertical transmission is a mechanism of *Bartonella* persistence (Kosoy et al., 1998).

Besides, *Bartonella* infection stimulates the secretion of certain cytokines in the host. Specifically, interferon γ, which activates macrophages to destroy intracellular pathogens, is highly expressed in the peripheral blood of cats with bacteremia (Kabeya et al., 2009) and in the spleen cells of *B. henselae*-infected mice (Kabeya et al., 2007). Additionally, innate immune defense mechanisms, such as mannan-binding proteins, may also play a role in the recognition and elimination of *B. henselae* (Ezekowitz and Stahl, 1988; Rodriguez-Barradas et al., 1995). In contrast, cell-mediated immune response (CMI) is crucial for eliminating pathogens when *Bartonella*, especially *B. henselae*, enters host cells (excluding erythrocytes) (Kabeya et al., 2009).

## *Bartonella* immune escape in mammalian host

*Bartonella* has also developed several strategies to evade the host’s immune response. Evasion of innate immune response is a prerequisite for *Bartonella* to establish intracellular infection in the host. When *Bartonella* is inoculated at subcutaneous or intradermal sites, it is first and foremost exposed to resident DCs and macrophages. *B. tribocorum* has been reported to be resistant to phagocytosis in rats (Hong et al., 2017). The phagocytosis resistance is likely delivered by the structural mechanism of bacterial aggregates and is validated by the fact that a *badA* knock-out *Bartonella* strain is more susceptible to phagocytosis by macrophages (Riess et al., 2004). Even if engulfed by macrophages, *Bartonella* can inhibit pyroptosis and suppress the expansion of the inflammatory response (Hong et al., 2017). Alternatively, it can form a unique *Bartonella*-containing vacuole (BCV) that can delay lysosomal targeting and destruction (Kyme et al., 2005).

As gram-negative bacteria, Bartonellae possess a lipopolysaccharide (LPS) component on the outer membrane that is a well-known ligand for Toll-like receptor 4 (TLR4). However, *Bartonella* LPS is poorly recognized by TLR4, which may also contribute to the low efficiency of phagocytosis. LPS of *B. quintana* and *B. henselae* exhibits several unusual features, including a unique structure, lipid A with a long fatty acid side chain, and a lack of an O-chain polysaccharide (Zahringer et al., 2004; Popa et al., 2007; Malgorzata-Miller et al., 2016). Furthermore, the LPS of *B. quintana* is an antagonist of TLR4, inhibiting the expression of cytokines, including IL-1β, IL-6, and tumor necrosis factor α, generated by TLR4-linked pathways (Popa et al., 2007; Mosepele et al., 2012; Malgorzata-Miller et al., 2016). During *Bartonella* infection, TLR4 does not play a significant role in pathogen recognition. Instead, the host’s innate immune response is triggered through TLR2 recognition (Vermi et al., 2006; Matera et al., 2008). In the case of *Candida albicans* infection, TLR2 has been shown to induce IL-10 secretion and Treg cell survival, thereby inhibiting inflammation (Netea et al., 2004). However, it is still unknown whether *Bartonella* infection triggers a similar response.

An antagonistic mechanism against the complement system has also been identified in *Bartonella*. BadA is proven to be critical for bacterial resistance to the host complement system. A *badA*-knockout *Bartonella birtlesii* mutant was susceptible to mouse serum, whereas the wild-type *B. birtlesii* expressing active BadA was resistant, which could be neutralized by anti-BadA antibodies (Deng et al., 2012).

Antigenic variation is another efficient strategy for *Bartonella* to evade the host immune response (Figure 3). Two important virulence factors, BadA in *B. henselae* (Riess et al., 2004) and the Vomp family in *B. quintana* (Zhang et al., 2004), have great potential to evade the host immune response through antigenic variation. The stem domain of *badA* and *vomp* contains modular and repetitive DNA sequences that perhaps increase the recombination frequency of the corresponding genes (Linke et al., 2006), while the internal structure of the *vomp* locus promotes recombination and deletion of the *vomp* gene (Zhang et al., 2004). The variable expression of Vomp family members was detected in both macaque animal models and *B. quintana-*infected humans (Zhang et al., 2004), and the expression of BadA seemly exhibits the characterization of phase variation (Thibau et al., 2022a). Intriguingly, *B. bacilliformis* can evade Toll-like receptor 5 by possessing unique amino acid sequences in flagellin that are different from the evolutionarily conserved ones required for microbial fitness (Andersen-Nissen et al., 2005; Figure 3). Bartonella CFA, an important target of protective antibodies, has a hypervariable antigenic region in both human- and mouse-hosted *Bartonella* strains (Siewert et al., 2022b). This hypervariability has the potential to allow for antibody evasion in the same mammalian host infected with different *Bartonella* strains through multiple sequential or timewise overlapping modes.

Suppression of immune response is also employed by *Bartonella* to escape the host immune response. Some *Bartonella* species, such as *B. quintana* and *B. henselae*, can promote mononuclear cells and DCs to secrete IL-10 (Papadopoulos et al., 2001; Vermi et al., 2006; Foca et al., 2012; Schmidgen et al., 2014), which suppresses inflammation response and facilitates the continuous progression of asymptomatic pathogen infection (Kabeya et al., 2007; Couper et al., 2008). The vital function of IL-10 in *Bartonella*’s immune evasion has been demonstrated in mouse models, as the pathogen *B. birtlesii* is unable to establish bacteremia in IL-10 knockout mice (Marignac et al., 2010). What’s more, recent studies showed that BepD activated the STAT3 pathway and promoted the secretion of anti-inflammatory cytokine IL-10, which may play a role in the resistance of *Bartonella* to innate immune cells in the dermal niche (Fromm and Dehio, 2021). In addition, *B. vinsonii* reduces MHC-II expression on the surface of B cells in dogs, suggesting the declined B-cell antigen presentation to helper T cells (Pappalardo et al., 2001). In cats, post-infection with *B. henselae* leads to a decreased number of CD4^+^ cells (Kabeya et al., 2009), yet the corresponding mechanism is still unknown.

The intracellular persistence of *Bartonella* is another unique strategy to avoid the host humoral immune system. *B. quintana*, for example, colonizes intracellularly without causing hemolysis of erythrocytes, which benefits the spread through body lice and cause repeated infection (Rolain et al., 2002, 2003). Recent studies have also detected *B. quintana* in human dental pulp stem cells (DPSCs), and the increase of bacterial load within the cellular niche does not affect the proliferation of DPSCs (Oumarou Hama et al., 2021).

The host immune response and *Bartonella* immune escape competes and restricts each other as shown in Figure 3. A strong immune response can reduce the frequency of host bacteremia, leading to recovery under the combined action of specific and non-specific immunity. However, weak immune response can result in repeated and long-term *Bartonella* infection, which can even be life-threatening. Therefore, studying the host immune response after *Bartonella* infection can help understand the causes of bacillary angiomatosis, peliosis hepatis, CSD, and other diseases, and provide new ideas for alleviating the clinical symptoms of patients after *Bartonella* infection.

## Conclusion

*Bartonella* is primarily transmitted through the feces of blood-sucking arthropods or traumatic contact with infected animals. The invasion of Bartonellae in lineage 3 and lineage 4 is facilitated by the VirB/VirD4 T4SS system and Beps protein. The pathogen’s invasion triggers the humoral and cellular immune responses of the host. Particularly, the immune system produces various antibodies to neutralize pathogens, playing a vital role in the removal of *Bartonella* from the bloodstream.

However, *Bartonella* species have developed several mechanisms to evade or resist the host’s immune response, which allow them to cause long-termed and repeated bacteremia. Some *Bartonella* species rely on special LPS structure or are hidden in host cells, while others stimulate the host to produce cytokines that weaken the immune response. Here we briefly summarized the recent findings on how *Bartonella* interacts with host ECs. Among the well-studied molecules are the trimeric family of autotransporters, α-enolase, and BafA. Additionally, *Bartonella* infection of MSCs can increase the susceptibility of ECs to *Bartonella*. The molecular involvement during erythrocyte invasion has also deepened our understanding of *Bartonella* infection. Investigating the mechanism of *Bartonella* infection on ECs and erythrocytes is crucial for comprehending *Bartonella* disease. Despite significant efforts, there remain numerous uncertain aspects that require further investigation, such as fully elucidating the functions of various types of molecules mentioned above. Future studies on *Bartonella* may require more appropriate *in vitro* and *in vivo* infection models, as well as functional genomics studies. Our efforts would be directed toward developing more effective and economical methods for detecting *Bartonella* in the population, as well as preventing and treating the diseases caused by *Bartonella.*

## Author contributions

TL and JF contributed to the conception of the review and reviewed and revised the manuscript. XJ, YG, TL, JF, YX, JL, and JS collected and organized the data. XJ and YG wrote the manuscript. All authors contributed to the article and approved the submitted version.

## References

[B1] Abbott R. C. Chomel B. B. Kasten R. W. Floyd-Hawkins K. A. Kikuchi Y. Koehler J. E. (1997). Experimental and natural infection with Bartonella henselae in domestic cats. *Comp. Immunol. Microbiol. Infect. Dis*. 20 41–51. 9023040 10.1016/s0147-9571(96)00025-2

[B2] Andersen-Nissen E. Smith K. D. Strobe K. L. Barrett S. L. R. Cookson B. T. Logan S. M. (2005). Evasion of Toll-like receptor 5 by flagellated bacteria. *Proc. Natl. Acad. Sci. U.S.A*. 102 9247–9252. 15956202 10.1073/pnas.0502040102 PMC1166605

[B3] Battisti J. M. Lawyer P. G. Minnick M. F. (2015). Colonization of Lutzomyia verrucarum and Lutzomyia longipalpis sand flies (Diptera: Psychodidae) by Bartonella bacilliformis, the etiologic agent of carrion’s disease. *PLoS Negl. Trop. Dis.* 9:e0004128. 10.1371/journal.pntd.0004128 26436553 PMC4593541

[B4] Biancardi A. L. Curi A. L. (2014). Cat-scratch disease. *Ocul. Immunol. Inflamm.* 22 148–154. 10.3109/09273948.2013.833631 24107122

[B5] Bown K. J. Bennet M. Begon M. (2004). Flea-borne Bartonella grahamii and *Bartonella taylorii* in bank voles. *Emerg. Infect. Dis*. 10 684–687. 15200860 10.3201/eid1004.030455 PMC3323072

[B6] Breitschwerdt E. B. Maggi R. G. Chomel B. B. Lappin M. R. (2010a). Bartonellosis: an emerging infectious disease of zoonotic importance to animals and human beings. *J. Vet. Emerg. Crit. Care* 20 8–30. 10.1111/j.1476-4431.2009.00496.x 20230432

[B7] Breitschwerdt E. B. Maggi R. G. Farmer P. Mascarelli P. E. (2010b). Molecular evidence of perinatal transmission of Bartonella vinsonii subsp. berkhoffii and Bartonella henselae to a child. *J. Clin. Microbiol.* 48 2289–2293. 10.1128/JCM.00326-10 20392912 PMC2884525

[B8] Brenner D. J. O’Connor S. P. Winkler H. H. Steigerwalt A. G. (1993). Proposals to unify the genera Bartonella and Rochalimaea, with descriptions of Bartonella quintana comb. nov., Bartonella vinsonii comb. nov., Bartonella henselae comb. nov., and Bartonella elizabethae comb. nov., and to remove the family Bartonellaceae from the order Rickettsiales. *Int. J. Syst. Bacteriol.* 43 777–786. 10.1099/00207713-43-4-777 8240958

[B200] Byam W. Lloyd L. (1920). Trench fever: its epidemiology and endemiology. *Proc. R. Soc. Med*. 13, 1–27. 10.1177/003591572001301501 PMC2152678 19981284

[B9] Cappello P. Principe M. Bulfamante S. Novelli F. (2017). Alpha-Enolase (ENO1), a potential target in novel immunotherapies. *Front. Biosci.* 22:944–959. 10.2741/4526 27814656

[B10] Cascales E. Christie P. J. (2003). The versatile bacterial type IV secretion systems. *Nat. Rev. Microbiol.* 1 137–149. 10.1038/nrmicro753 15035043 PMC3873781

[B11] Chamberlin J. Laughlin L. W. Romero S. Solórzano N. Gordon S. Andre R. G. (2002). Epidemiology of endemic Bartonella bacilliformis: a prospective cohort study in a Peruvian mountain valley community. *J. Infect. Dis*. 186 983–990. 12232839 10.1086/344054

[B12] Chang C. C. Lee C. J. Ou L. S. Wang C. J. Huang Y. C. (2016). Disseminated cat-scratch disease: case report and review of the literature. *Paediatr. Int. Child Health* 36 232–234. 10.1179/2046905515Y.0000000005 25940800

[B13] Chenoweth M. R. Greene C. E. Krause D. C. Gherardini F. C. (2004). Predominant outer membrane antigens of Bartonella henselae. *Infect. Immun.* 72 3097–3105. 10.1128/IAI.72.6.3097-3105.2004 15155610 PMC415646

[B14] Chomel B. B. Boulouis H. J. Breitschwerdt E. B. Kasten R. W. Vayssier-Taussat M. Birtles R. J. (2009). Ecological fitness and strategies of adaptation of Bartonella species to their hosts and vectors. *Vet. Res.* 40:29. 10.1051/vetres/2009011 19284965 PMC2695021

[B15] Chomel B. B. Kasten R. W. Floyd-Hawkins K. Chi B. Yamamoto K. Roberts-Wilson J. (1996). Experimental transmission of Bartonella henselae by the cat flea. *J. Clin. Microbiol.* 34 1952–1956. 8818889 10.1128/jcm.34.8.1952-1956.1996 PMC229161

[B16] Clemente N. S. Ugarte-Gil C. Solorzano N. Maguiña C. Moore D. (2016). An outbreak of Bartonella bacilliformis in an endemic andean community. *PLoS One*. 11:e0150525. 10.1371/journal.pone.0150525 26991495 PMC4798250

[B17] Coleman S. A. Minnick M. F. (2001). Establishing a direct role for the Bartonella bacilliformis invasion-associated locus B (IalB) protein in human erythrocyte parasitism. *Infect. Immun.* 69 4373–4381. 11401976 10.1128/IAI.69.7.4373-4381.2001 PMC98509

[B18] Cotte V. Bonnet S. Le Rhun D. Le Naour E. Chauvin A. Boulouis H. J. (2008). Transmission of Bartonella henselae by Ixodes ricinus. *Emerg. Infect. Dis.* 14 1074–1080. 10.3201/eid1407.071110 18598628 PMC2600320

[B19] Couper K. N. Blount D. G. Riley E. M. (2008). IL-10: the master regulator of immunity to infection. *J. Immunol.* 180 5771–5777. 10.4049/jimmunol.180.9.5771 18424693

[B20] Dehio C. (2005). Bartonella–host-cell interactions and vascular tumour formation. *Nat. Rev. Microbiol.* 3 621–631. 10.1038/nrmicro1209 16064054

[B21] Dehio C. (2008). Infection-associated type IV secretion systems of Bartonella and their diverse roles in host cell interaction. *Cell. Microbiol.* 10 1591–1598. 10.1111/j.1462-5822.2008.01171.x 18489724 PMC2610397

[B22] Dehio C. Tsolis R. M. (2017). Type IV effector secretion and subversion of host functions by Bartonella and Brucella species. *Curr. Top. Microbiol. Immunol.* 413 269–295. 10.1007/978-3-319-75241-9_11 29536363

[B23] Deng H. Le Rhun D. Buffet J. P. Cotte V. Read A. Birtles R. J. (2012). Strategies of exploitation of mammalian reservoirs by Bartonella species. *Vet. Res.* 43:15. 10.1186/1297-9716-43-15 22369683 PMC3430587

[B24] Deng H. Pang Q. Xia H. Le Rhun D. Le Naour E. Yang C. (2016). Identification and functional analysis of invasion associated locus B (IalB) in Bartonella species. *Microb. Pathog.* 98 171–177. 10.1016/j.micpath.2016.05.007 27515099

[B25] Deng H. Wu S. Song Q. Zhang J. Sang F. Sun X. (2019). Cloning and identification of Bartonella alpha-enolase as a plasminogen-binding protein. *Microb. Pathog.* 135:103651. 10.1016/j.micpath.2019.103651 31398532

[B26] Díaz-Ramos A. Roig-Borrellas A. García-Melero A. López-Alemany R. (2012). α-Enolase, a multifunctional protein: its role on pathophysiological situations. *J. Biomed. Biotechnol.* 2012 156795. 10.1155/2012/156795 23118496 PMC3479624

[B27] do Amaral R. B. Cardozo M. V. Varani A. M. Furquim M. E. C. Dias C. M. Assis W. O. (2022a). First report of Bartonella spp. in marsupials from Brazil, with a description of Bartonella harrusi sp. nov. and a new proposal for the taxonomic reclassification of species of the Genus Bartonella. *Microorganisms* 10:1609. 10.3390/microorganisms10081609 36014025 PMC9414547

[B28] do Amaral R. B. Cardozo M. V. Varani A. M. Goncalves L. R. Furquim M. E. C. Dias C. M. (2022b). Bartonella machadoae sp. nov. isolated from wild rodents in the Pantanal wetland. *Acta Trop.* 229:106368. 10.1016/j.actatropica.2022.106368 35157842

[B29] Duscher G. G. Hodzic A. Potkonjak A. Leschnik M. W. Spergser J. (2018). Bartonella henselae and rickettsia felis detected in cat fleas (Ctenocephalides felis) derived from Eastern Austrian Cats. *Vector Borne Zoonotic Dis* 18 282–284. 10.1089/vbz.2017.2215 29668398

[B30] Eicher S. C. Dehio C. (2012). Bartonella entry mechanisms into mammalian host cells. *Cell. Microbiol.* 14 1166–1173. 10.1111/j.1462-5822.2012.01806.x 22519749

[B31] Ezekowitz R. A. Stahl P. D. (1988). The structure and function of vertebrate mannose lectin-like proteins. *J. Cell Sci. Suppl*. 9 121–133. 3077136 10.1242/jcs.1988.supplement_9.6

[B32] Foca A. Liberto M. C. Quirino A. Matera G. (2012). Lipopolysaccharides: from Erinyes to Charites. *Mediators Inflamm.* 2012:684274. 10.1155/2012/684274 22665953 PMC3361297

[B33] Foil L. Andress E. Freeland R. L. Roy A. F. Rutledge R. Triche P. C. (1998). Experimental infection of domestic cats with Bartonella henselae by inoculation of Ctenocephalides felis (Siphonaptera: Pulicidae) feces. *J. Med. Entomol.* 35 625–628. 10.1093/jmedent/35.5.625 9775583

[B34] Frank A. C. Alsmark C. M. Thollesson M. Andersson S. G. (2005). Functional divergence and horizontal transfer of type IV secretion systems. *Mol. Biol. Evol.* 22 1325–1336. 10.1093/molbev/msi124 15746011

[B35] Fromm K. Dehio C. (2021). The impact of Bartonella VirB/VirD4 Type IV secretion system effectors on eukaryotic host cells. *Front. Microbiol.* 12:762582. 10.3389/fmicb.2021.762582 34975788 PMC8714903

[B36] Gai M. d’Onofrio G. di Vico M. C. Ranghino A. Nappo A. Diena D. (2015). Cat-scratch disease: case report and review of the literature. *Transplant Proc.* 47 2245–2247. 10.1016/j.transproceed.2015.07.014 26361690

[B37] Garcia-Quintanilla M. Dichter A. A. Guerra H. Kempf V. A. J. (2019). Carrion’s disease: more than a neglected disease. *Parasit Vectors* 12 141. 10.1186/s13071-019-3390-2 30909982 PMC6434794

[B38] Gilmore R. D. Jr. Bellville T. M. Sviat S. L. Frace M. (2005). The Bartonella vinsonii subsp. arupensis immunodominant surface antigen BrpA gene, encoding a 382-kilodalton protein composed of repetitive sequences, is a member of a multigene family conserved among Bartonella species. *Infect. Immun.* 73 3128–3136. 10.1128/IAI.73.5.3128-3136.2005 15845521 PMC1087387

[B39] Gomes C. Martinez-Puchol S. Ruiz-Roldan L. Pons M. J. Del Valle Mendoza J. Ruiz J. (2016a). Development and characterisation of highly antibiotic resistant Bartonella bacilliformis mutants. *Sci. Rep.* 6:33584. 10.1038/srep33584 27667026 PMC5035977

[B40] Gomes C. Palma N. Pons M. J. Magallon-Tejada A. Sandoval I. Tinco-Valdez C. (2016b). Succinyl-CoA synthetase: new antigen candidate of Bartonella bacilliformis. *PLoS Negl. Trop. Dis.* 10:e0004989. 10.1371/journal.pntd.0004989 27627803 PMC5023120

[B41] Goncalves-Oliveira J. Gutierrez R. Schlesener C. L. Jaffe D. A. Aguilar-Setien A. Boulouis H. J. (2023). Genomic characterization of three novel Bartonella strains in a rodent and two bat species from Mexico. *Microorganisms* 11:340. 10.3390/microorganisms11020340 36838305 PMC9962129

[B42] Gundi V. A. Davoust B. Khamis A. Boni M. Raoult D. La Scola B. (2004). Isolation of Bartonella rattimassiliensis sp. nov. and Bartonella phoceensis sp. nov. from European Rattus norvegicus. *J. Clin. Microbiol.* 42 3816–3818. 10.1128/JCM.42.8.3816-3818.2004 15297537 PMC497573

[B43] Gutierrez R. Shalit T. Markus B. Yuan C. Nachum-Biala Y. Elad D. (2020). Bartonella kosoyi sp. nov. and Bartonella krasnovii sp. nov., two novel species closely related to the zoonotic Bartonella elizabethae, isolated from black rats and wild desert rodent-fleas. *Int. J. Syst. Evol. Microbiol.* 70 1656–1665. 10.1099/ijsem.0.003952 32100689

[B44] Harms A. Dehio C. (2012). Intruders below the radar: molecular pathogenesis of Bartonella spp. *Clin. Microbiol. Rev.* 25 42–78. 10.1128/CMR.05009-11 22232371 PMC3255967

[B45] Harms A. Segers F. H. Quebatte M. Mistl C. Manfredi P. Korner J. (2017). Evolutionary dynamics of pathoadaptation revealed by three independent acquisitions of the VirB/D4 type IV secretion system in Bartonella. *Genome Biol. Evol.* 9 761–776. 10.1093/gbe/evx042 28338931 PMC5381568

[B46] Hendrix L. R. Kiss K. (2003). Studies on the identification of deforming factor from Bartonella bacilliformis. *Ann. N.Y. Acad. Sci.* 990 596–604. 10.1111/j.1749-6632.2003.tb07433.x 12860696

[B47] Higgins J. A. Radulovic S. Jaworski D. C. Azad A. F. (1996). Acquisition of the cat scratch disease agent Bartonella henselae by cat fleas (Siphonaptera:Pulicidae). *J. Med. Entomol.* 33 490–495. 10.1093/jmedent/33.3.490 8667399

[B49] Hong J. Li Y. Hua X. Bai Y. Wang C. Zhu C. (2017). Lymphatic circulation disseminates Bartonella infection into bloodstream. *J. Infect. Dis.* 215 303–311. 10.1093/infdis/jiw526 27803173

[B50] Iwaki-Egawa S. Ihler G. M. (1997). Comparison of the abilities of proteins from Bartonella bacilliformis and Bartonella henselae to deform red cell membranes and to bind to red cell ghost proteins. *FEMS Microbiol. Lett.* 157 207–217. 10.1111/j.1574-6968.1997.tb12775.x 9418257

[B51] Kabeya H. Umehara T. Okanishi H. Tasaki I. Kamiya M. Misawa A. (2009). Experimental infection of cats with Bartonella henselae resulted in rapid clearance associated with T helper 1 immune responses. *Microbes Infect.* 11 716–720. 10.1016/j.micinf.2009.03.008 19348961

[B52] Kabeya H. Yamasaki A. Ikariya M. Negishi R. Chomel B. B. Maruyama S. (2007). Characterization of Th1 activation by Bartonella henselae stimulation in BALB/c mice: inhibitory activities of interleukin-10 for the production of interferon-gamma in spleen cells. *Vet. Microbiol.* 119 290–296. 10.1016/j.vetmic.2006.08.010 17005337

[B54] Kaiser P. O. Riess T. Wagner C. L. Linke D. Lupas A. N. Schwarz H. (2008). The head of Bartonella adhesin A is crucial for host cell interaction of Bartonella henselae. *Cell. Microbiol.* 10 2223–2234. 10.1111/j.1462-5822.2008.01201.x 18627378

[B201] Kempf V. A. Lebiedziejewski M. Alitalo K. Wälzlein J. H. Ehehalt U. Fiebig J. (2005). Activation of hypoxia-inducible factor-1 in bacillary angiomatosis: evidence for a role of hypoxia-inducible factor-1 in bacterial infections. *Circulation* 111, 1054–1062. 10.1161/01.CIR.0000155608.07691.B 15723970

[B55] Kempf V. A. Volkmann B. Schaller M. Sander C. A. Alitalo K. Riess T. (2001). Evidence of a leading role for VEGF in Bartonella henselae-induced endothelial cell proliferations. *Cell. Microbiol.* 3 623–632. 10.1046/j.1462-5822.2001.00144.x 11553014

[B56] Keragala C. B. Medcalf R. L. (2021). Plasminogen: an enigmatic zymogen. *Blood* 137 2881–2889. 10.1182/blood.2020008951 33735914

[B58] Koesling J. Aebischer T. Falch C. Schulein R. Dehio C. (2001). Cutting edge: antibody-mediated cessation of hemotropic infection by the intraerythrocytic mouse pathogen Bartonella grahamii. *J. Immunol.* 167 11–14. 10.4049/jimmunol.167.1.11 11418625

[B59] Kosoy M. Y. Regnery R. L. Kosaya O. I. Jones D. C. Marston E. L. Childs J. E. (1998). Isolation of Bartonella spp. from embryos and neonates of naturally infected rodents. *J. Wildl. Dis.* 34 305–309. 9577777 10.7589/0090-3558-34.2.305

[B60] Kostrzewski J. (1950). [Epidemiology of trench fever]. *Medycyna Doswiadczalna I Mikrobiologia* 2 19–51. 14806013

[B61] Krol N. Militzer N. Stobe E. Nijhof A. M. Pfeffer M. Kempf V. A. J. (2021). Evaluating transmission paths for three different Bartonella spp. in ixodes ricinus ticks using artificial feeding. *Microorganisms* 9:901. 10.3390/microorganisms9050901 33922378 PMC8146832

[B62] Kyme P. A. Haas A. Schaller M. Peschel A. Iredell J. Kempf V. A. (2005). Unusual trafficking pattern of Bartonella henselae -containing vacuoles in macrophages and endothelial cells. *Cell. Microbiol.* 7 1019–1034. 10.1111/j.1462-5822.2005.00531.x 15953033

[B202] Lähteenmäki K. Kuusela P. Korhonen T. K. (2001). Bacterial plasminogen activators and receptors. *FEMS Microbiol. Rev*. 25, 531–552. 10.1111/j.1574-6976.2001.tb00590.x 11742690

[B63] Leibler J. H. Zakhour C. M. Gadhoke P. Gaeta J. M. (2016). Zoonotic and vector-borne infections among urban homeless and marginalized people in the United States and Europe, 1990-2014. *Vector Borne Zoonotic Dis.* 16 435–444. 10.1089/vbz.2015.1863 27159039

[B64] Linke D. Riess T. Autenrieth I. B. Lupas A. Kempf V. A. (2006). *Trimeric autotransporter adhesin*s: variable structure, common function. *Trends Microbiol.* 14 264–270. 10.1016/j.tim.2006.04.005 16678419

[B65] Litwin C. M. Johnson J. M. (2005). Identification, cloning, and expression of the CAMP-like factor autotransporter gene (cfa) of Bartonella henselae. *Infect. Immun.* 73 4205–4213. 10.1128/IAI.73.7.4205-4213.2005 15972511 PMC1168562

[B66] Liu M. Biville F. (2013). Managing iron supply during the infection cycle of a flea borne pathogen, Bartonella henselae. *Front. Cell. Infect. Microbiol.* 3:60. 10.3389/fcimb.2013.00060 24151576 PMC3799009

[B67] Liu Q. Eremeeva M. E. Li D. (2012). Bartonella and Bartonella infections in China: from the clinic to the laboratory. *Comp. Immunol. Microbiol. Infect. Dis.* 35 93–102. 10.1016/j.cimid.2012.01.002 22304899

[B68] Liu Y. Chen J. Lang H. Zheng H. (2022). Bartonella choladocola sp. nov. and Bartonella apihabitans sp. nov., two novel species isolated from honey bee gut. *Syst. Appl. Microbiol.* 45:126372. 10.1016/j.syapm.2022.126372 36279689

[B69] Lu Y. Y. Franz B. Truttmann M. C. Riess T. Gay-Fraret J. Faustmann M. (2013). Bartonella henselae *trimeric autotransporter adhesin* BadA expression interferes with effector translocation by the VirB/D4 type IV secretion system. *Cell Microbiol.* 15 759–778. 10.1111/cmi.12070 23163798

[B70] Maguina C. Guerra H. Ventosilla P. (2009). Bartonellosis. *Clin. Dermatol.* 27 271–280. 10.1016/j.clindermatol.2008.10.006 19362689

[B71] Malgorzata-Miller G. Heinbockel L. Brandenburg K. van der Meer J. W. Netea M. G. Joosten L. A. (2016). Bartonella quintana lipopolysaccharide (LPS): structure and characteristics of a potent TLR4 antagonist for in-vitro and in-vivo applications. *Sci. Rep.* 6:34221. 10.1038/srep34221 27670746 PMC5037446

[B203] Mändle T. Einsele H. Schaller M. Neumann D. Vogel W. Autenrieth I. B. (2005). Infection of human CD34+ progenitor cells with Bartonella henselae results in intraerythrocytic presence of *B. henselae. Blood* 106, 1215–1222. 10.1182/blood-2004-12-4670 15860668

[B72] Marignac G. Barrat F. Chomel B. Vayssier-Taussat M. Gandoin C. Bouillin C. (2010). Murine model for Bartonella birtlesii infection: new aspects. *Comp. Immunol. Microbiol. Infect. Dis.* 33 95–107. 10.1016/j.cimid.2008.07.011 20097421

[B73] Marlaire S. Dehio C. (2021). Bartonella effector protein C mediates actin stress fiber formation via recruitment of GEF-H1 to the plasma membrane. *PLoS Pathog.* 17:e1008548. 10.1371/journal.ppat.1008548 33508040 PMC7842960

[B74] Matera G. Liberto M. C. Joosten L. A. Vinci M. Quirino A. Pulicari M. C. (2008). The Janus face of Bartonella quintana recognition by Toll-like receptors (TLRs): a review. *Eur. Cytokine Netw.* 19 113–118. 10.1684/ecn.2008.0128 18775802

[B75] McCord A. M. Resto-Ruiz S. I. Anderson B. E. (2006). Autocrine role for interleukin-8 in Bartonella henselae-induced angiogenesis. *Infect. Immun.* 74 5185–5190. 10.1128/IAI.00622-06 16926411 PMC1594831

[B76] McGill S. L. Regnery R. L. Karem K. L. (1998). Characterization of human immunoglobulin (Ig) isotype and IgG subclass response to Bartonella henselae infection. *Infect. Immun.* 66 5915–5920. 10.1128/IAI.66.12.5915-5920.1998 9826373 PMC108749

[B77] Mernaugh G. Ihler G. M. (1992). Deformation factor: an extracellular protein synthesized by Bartonella bacilliformis that deforms erythrocyte membranes. *Infect. Immun.* 60 937–943. 10.1128/iai.60.3.937-943.1992 1541567 PMC257577

[B78] Minnick M. F. Anderson B. E. Lima A. Battisti J. M. Lawyer P. G. Birtles R. J. (2014). Oroya fever and verruga peruana: bartonelloses unique to South America. *PLoS Negl. Trop. Dis.* 8:e2919. 10.1371/journal.pntd.0002919 25032975 PMC4102455

[B79] Mitchell S. J. Minnick M. F. (1995). Characterization of a two-gene locus from Bartonella bacilliformis associated with the ability to invade human erythrocytes. *Infect. Immun.* 63 1552–1562. 10.1128/iai.63.4.1552-1562.1995 7890422 PMC173188

[B80] Mosepele M. Mazo D. Cohn J. (2012). Bartonella infection in immunocompromised hosts: immunology of vascular infection and vasoproliferation. *Clin. Dev. Immunol.* 2012:612809. 10.1155/2012/612809 22162717 PMC3227422

[B81] Muller N. F. Kaiser P. O. Linke D. Schwarz H. Riess T. Schafer A. (2011). *Trimeric autotransporter adhesin*-dependent adherence of Bartonella henselae, Bartonella quintana, and Yersinia enterocolitica to matrix components and endothelial cells under static and dynamic flow conditions. *Infect. Immun.* 79 2544–2553. 10.1128/IAI.01309-10 21536788 PMC3191982

[B82] Musso T. Badolato R. Ravarino D. Stornello S. Panzanelli P. Merlino C. (2001). Interaction of Bartonella henselae with the murine macrophage cell line J774: infection and proinflammatory response. *Infect. Immun.* 69 5974–5980. 10.1128/IAI.69.10.5974-5980.2001 11553533 PMC98724

[B83] Netea M. G. Sutmuller R. Hermann C. Van der Graaf C. A. Van der Meer J. W. van Krieken J. H. (2004). Toll-like receptor 2 suppresses immunity against Candida albicans through induction of IL-10 and regulatory T cells. *J. Immunol.* 172 3712–3718. 10.4049/jimmunol.172.6.3712 15004175

[B84] O’Rourke F. Mändle T. Urbich C. Dimmeler S. Michaelis U. R. Brandes R. P. (2015). Reprogramming of myeloid angiogenic cells by Bartonella henselae leads to microenvironmental regulation of pathological angiogenesis. *Cell Microbiol.* 17 1447–1463. 10.1111/cmi.12447 25857345

[B85] Okaro U. Addisu A. Casanas B. Anderson B. (2017). Bartonella species, an emerging cause of blood-culture-negative endocarditis. *Clin. Microbiol. Rev.* 30 709–746. 10.1128/CMR.00013-17 28490579 PMC5475225

[B86] Okaro U. George S. Anderson B. (2021). What is in a cat scratch? Growth of Bartonella henselae in a biofilm. *Microorganisms* 9:835. 10.3390/microorganisms9040835 33919891 PMC8070961

[B87] Okujava R. Guye P. Lu Y. Y. Mistl C. Polus F. Vayssier-Taussat M. (2014). A translocated effector required for Bartonella dissemination from derma to blood safeguards migratory host cells from damage by co-translocated effectors. *PLoS Pathog.* 10:e1004187. 10.1371/journal.ppat.1004187 24945914 PMC4063953

[B88] Oskouizadeh K. Zahraei-Salehi T. Aledavood S. (2010). Detection of Bartonella henselae in domestic cats’ saliva. *Iran J. Microbiol*. 2 80–84. 22347553 PMC3279777

[B89] Oumarou Hama H. Hamada A. Aboudharam G. Ghigo E. Drancourt M. (2021). Human dental pulp stem cells: a sanctuary for relapsing Bartonella quintana. *Microb. Pathog.* 153:104797. 10.1016/j.micpath.2021.104797 33609646

[B90] Papadopoulos N. G. Gourgiotis D. Bossios A. Fretzayas A. Moustaki M. Karpathios T. (2001). Circulating cytokines in patients with cat scratch disease. *Clin. Infect. Dis.* 33 e54–56. 11512109 10.1086/322596

[B91] Pappalardo B. L. Brown T. T. Tompkins M. Breitschwerdt E. B. (2001). Immunopathology of Bartonella vinsonii (berkhoffii) in experimentally infected dogs. *Vet. Immunol. Immunopathol*. 83 125–147. 11730925 10.1016/s0165-2427(01)00372-5

[B92] Patten J. Wang K. (2021). Fibronectin in development and wound healing. *Adv. Drug Deliv. Rev.* 170 353–368. 10.1016/j.addr.2020.09.005 32961203

[B93] Pitassi L. H. de Paiva Diniz P. P. Scorpio D. G. Drummond M. R. Lania B. G. Barjas-Castro M. L. (2015). Bartonella spp. bacteremia in blood donors from Campinas. *Brazil. PLoS Negl. Trop. Dis.* 9:e0003467. 10.1371/journal.pntd.0003467 25590435 PMC4295888

[B94] Pons M. J. Gomes C. Aguilar R. Barrios D. Aguilar-Luis M. A. Ruiz J. (2017). Immunosuppressive and angiogenic cytokine profile associated with Bartonella bacilliformis infection in post-outbreak and endemic areas of Carrion’s disease in Peru. *PLoS Negl. Trop. Dis.* 11:e0005684. 10.1371/journal.pntd.0005684 28628613 PMC5491314

[B95] Popa C. Abdollahi-Roodsaz S. Joosten L. A. Takahashi N. Sprong T. Matera G. (2007). Bartonella quintana lipopolysaccharide is a natural antagonist of Toll-like receptor 4. *Infect. Immun.* 75 4831–4837. 10.1128/IAI.00237-07 17606598 PMC2044526

[B96] Pulliainen A. T. Dehio C. (2012). Persistence of Bartonella spp. stealth pathogens: from subclinical infections to vasoproliferative tumor formation. *FEMS Microbiol. Rev.* 36 563–599. 10.1111/j.1574-6976.2012.00324.x 22229763

[B97] Pulliainen A. T. Pieles K. Brand C. S. Hauert B. Bohm A. Quebatte M. (2012). Bacterial effector binds host cell adenylyl cyclase to potentiate Galphas-dependent cAMP production. *Proc. Natl. Acad. Sci. U.S.A.* 109 9581–9586. 10.1073/pnas.1117651109 22635269 PMC3386119

[B98] Resto-Ruiz S. I. Schmiederer M. Sweger D. Newton C. Klein T. W. Friedman H. (2002). Induction of a potential paracrine angiogenic loop between human THP-1 macrophages and human microvascular endothelial cells during Bartonella henselae infection. *Infect. Immun.* 70 4564–4570. 10.1128/IAI.70.8.4564-4570.2002 12117969 PMC128175

[B99] Ribeiro J. M. (1995). Blood-feeding arthropods: live syringes or invertebrate pharmacologists? *Infect. Agents Dis.* 4 143–152. 8548192

[B100] Riess T. Andersson S. G. Lupas A. Schaller M. Schäfer A. Kyme P. (2004). *Bartonella* adhesin A mediates a proangiogenic host cell response. *J. Exp. Med.* 200 1267–1278. 10.1084/jem.20040500 15534369 PMC2211922

[B101] Rodriguez-Barradas M. C. Bandres J. C. Hamill R. J. Trial J. Clarridge J. E. Baughn R. E. (1995). In vitro evaluation of the role of humoral immunity against Bartonella henselae. *Infect. Immun*. 63 2367–2370. 7768623 10.1128/iai.63.6.2367-2370.1995 PMC173313

[B102] Rolain J. M. Brouqui P. Koehler J. E. Maguina C. Dolan M. J. Raoult D. (2004). Recommendations for treatment of human infections caused by Bartonella species. *Antimicrob. Agents Chemother.* 48 1921–1933. 10.1128/aac.48.6.1921-1933.2004 15155180 PMC415619

[B103] Rolain J. M. Foucault C. Guieu R. La Scola B. Brouqui P. Raoult D. (2002). Bartonella quintana in human erythrocytes. *Lancet* 360 226–228. 10.1016/s0140-6736(02)09462-x 12133660

[B104] Rolain J. M. Maurin M. Mallet M. N. Parzy D. Raoult D. (2003). Culture and antibiotic susceptibility of Bartonella quintana in human erythrocytes. *Antimicrob. Agents Chemother.* 47 614–619. 10.1128/AAC.47.2.614-619.2003 12543668 PMC151782

[B105] Sanchez Clemente N. Ugarte-Gil C. A. Solorzano N. Maguina C. Pachas P. Blazes D. (2012). Bartonella bacilliformis: a systematic review of the literature to guide the research agenda for elimination. *PLoS Negl. Trop. Dis.* 6:e1819. 10.1371/journal.pntd.0001819 23145188 PMC3493376

[B106] Sasaki T. Adachi T. Itoh K. Matsuoka M. Yamagishi T. Hirao M. (2021). Detection of Bartonella quintana Infection among the homeless population in Tokyo, Japan, from 2013-2015. *Jpn J. Infect. Dis.* 74 411–415. 10.7883/yoken.JJID.2020.505 33518618

[B107] Schmidgen T. Kaiser P. O. Ballhorn W. Franz B. Gottig S. Linke D. (2014). Heterologous expression of Bartonella adhesin A in *Escherichia coli* by exchange of *trimeric autotransporter adhesin* domains results in enhanced adhesion properties and a pathogenic phenotype. *J. Bacteriol.* 196 2155–2165. 10.1128/JB.01461-13 24682330 PMC4054196

[B108] Scutera S. Mitola S. Sparti R. Salvi V. Grillo E. Piersigilli G. (2021). Bartonella henselae persistence within mesenchymal stromal cells enhances endothelial cell activation and infectibility that amplifies the angiogenic process. *Infect. Immun.* 89:e0014121. 10.1128/IAI.00141-21 34031126 PMC8284938

[B109] Segers F. H. I. D. Kešnerová L. Kosoy M. Engel P. (2017). Genomic changes associated with the evolutionary transition of an insect gut symbiont into a blood-borne pathogen. *ISME J.* 11 1232–1244. 10.1038/ismej.2016.201 28234349 PMC5437933

[B110] Serafim T. D. Coutinho-Abreu I. V. Dey R. Kissinger R. Valenzuela J. G. Oliveira F. (2021). Leishmaniasis: the act of transmission. *Trends Parasitol.* 37 976–987. 10.1016/j.pt.2021.07.003 34389215

[B111] Siamer S. Dehio C. (2015). New insights into the role of Bartonella effector proteins in pathogenesis. *Curr. Opin. Microbiol.* 23 80–85. 10.1016/j.mib.2014.11.007 25461577

[B112] Siewert L. K. Dehio C. Pinschewer D. D. (2022a). Adaptive immune defense prevents Bartonella persistence upon trans-placental transmission. *PLoS Pathog.* 18:e1010489. 10.1371/journal.ppat.1010489 35580143 PMC9113594

[B113] Siewert L. K. Korotaev A. Sedzicki J. Fromm K. Pinschewer D. D. Dehio C. (2022b). Identification of the Bartonella autotransporter CFA as a protective antigen and hypervariable target of neutralizing antibodies in mice. *Proc. Natl. Acad. Sci. U.S.A.* 119:e2202059119. 10.1073/pnas.2202059119 35714289 PMC9231624

[B114] Sorg I. Schmutz C. Lu Y. Y. Fromm K. Siewert L. K. Bogli A. (2020). A Bartonella effector acts as signaling hub for intrinsic STAT3 activation to trigger anti-inflammatory responses. *Cell Host Microbe* 27 476–485.e7. 10.1016/j.chom.2020.01.015 32101706

[B115] Suzuki N. Kumadaki K. Tatematsu K. Doi Y. Tsukamoto K. (2023). The autotransporter BafA contributes to the proangiogenic potential of Bartonella elizabethae. *Microbiol. Immunol*. 67 248–257. 10.1111/1348-0421.13057 36810719

[B116] Tame J. R. (2011). Autotransporter protein secretion. *Biomol. Concepts* 2 525–536. 10.1515/bmc.2011.045 25962052

[B117] Thibau A. Hipp K. Vaca D. J. Chowdhury S. Malmstrom J. Saragliadis A. (2022a). Long-read sequencing reveals genetic adaptation of Bartonella Adhesin A among different Bartonella henselae isolates. *Front. Microbiol.* 13:838267. 10.3389/fmicb.2022.838267 35197960 PMC8859334

[B118] Thibau A. Vaca D. J. Bagowski M. Hipp K. Bender D. Ballhorn W. (2022b). Adhesion of Bartonella henselae to fibronectin is mediated via repetitive motifs present in the stalk of Bartonella Adhesin A. *Microbiol. Spectr.* 10:e0211722. 10.1128/spectrum.02117-22 36165788 PMC9602544

[B119] Truttmann M. C. Rhomberg T. A. Dehio C. (2011). Combined action of the type IV secretion effector proteins BepC and BepF promotes invasome formation of Bartonella henselae on endothelial and epithelial cells. *Cell. Microbiol.* 13 284–299. 10.1111/j.1462-5822.2010.01535.x 20964799

[B120] Tsukamoto K. Kumadaki K. Tatematsu K. Suzuki N. Doi Y. (2022). The passenger domain of Bartonella bacilliformis BafA promotes endothelial cell angiogenesis via the VEGF receptor signaling pathway. *mSphere* 7:e0008122. 10.1128/msphere.00081-22 35379004 PMC9044958

[B121] Tsukamoto K. Shinzawa N. Kawai A. Suzuki M. Kidoya H. Takakura N. (2020). The Bartonella autotransporter BafA activates the host VEGF pathway to drive angiogenesis. *Nat. Commun.* 11:3571. 10.1038/s41467-020-17391-2 32678094 PMC7366657

[B122] Vaca D. J. Thibau A. Leisegang M. S. Malmstrom J. Linke D. Eble J. A. (2022). Interaction of Bartonella henselae with fibronectin represents the molecular basis for adhesion to host cells. *Microbiol. Spectr.* 10:e0059822. 10.1128/spectrum.00598-22 35435766 PMC9241615

[B123] Vermi W. Facchetti F. Riboldi E. Heine H. Scutera S. Stornello S. (2006). Role of dendritic cell-derived CXCL13 in the pathogenesis of Bartonella henselae B-rich granuloma. *Blood* 107 454–462. 10.1182/blood-2005-04-1342 16189275

[B124] Wagner A. Dehio C. (2019). Role of distinct type-IV-secretion systems and secreted effector sets in host adaptation by pathogenic Bartonella species. *Cell. Microbiol.* 21:e13004. 10.1111/cmi.13004 30644157 PMC6519360

[B125] Windsor J. J. (2001). Cat-scratch disease: epidemiology, aetiology and treatment. *Br. J. Biomed. Sci.* 58 101–110. 11440202

[B126] Xu Y. H. Lu Z. Y. Ihler G. M. (1995). Purification of deformin, an extracellular protein synthesized by Bartonella bacilliformis which causes deformation of erythrocyte membranes. *Biochim. Biophys. Acta* 1234 173–183. 10.1016/0005-2736(94)00271-p 7696292

[B127] Zahringer U. Lindner B. Knirel Y. A. van den Akker W. M. Hiestand R. Heine H. (2004). Structure and biological activity of the short-chain lipopolysaccharide from Bartonella henselae ATCC 49882T. *J. Biol. Chem.* 279 21046–21054. 10.1074/jbc.M313370200 14766898

[B128] Zhang P. Chomel B. B. Schau M. K. Goo J. S. Droz S. Kelminson K. L. (2004). A family of variably expressed outer-membrane proteins (Vomp) mediates adhesion and autoaggregation in Bartonella quintana. *Proc. Natl. Acad. Sci. U.S.A.* 101 13630–13635. 10.1073/pnas.0405284101 15347808 PMC518805

