# Corrigendum: Advancements in understanding the molecular and immune mechanisms of *Bartonella* pathogenicity

**DOI:** 10.3389/fmicb.2023.1260035

**Published:** 2023-07-27

**Authors:** Xiaoxia Jin, Yuze Gou, Yuxian Xin, Jingwei Li, Jingrong Sun, Tingting Li, Jie Feng

**Affiliations:** ^1^Gansu Provincial Key Laboratory of Evidence Based Medicine and Clinical Translation and Lanzhou Center for Tuberculosis Research, School of Basic Medical Sciences, Lanzhou University, Lanzhou, China; ^2^Key Laboratory of Preclinical Study for New Drugs of Gansu Province, School of Basic Medical Sciences, Lanzhou, China; ^3^State Key Laboratory of Veterinary Etiological Biology, College of Veterinary Medicine, Lanzhou University, Lanzhou, China

**Keywords:** *Bartonella*, blood-sucking arthropods, endothelial cells, erythrocytes, antibody, immune escape

In the published article, there was an error in the section **Infection of endothelial cells (ECs)**, paragraph 1. The citation in “*Bartonella* species have been found to inhabit various host cells, including mononuclear phagocytes, CD34^+^ progenitor cells, and mesenchymal stromal cells (MSCs) (Scutera et al., 2021), but …” was incorrect. The correct citation is “(Mändle et al., 2005)”. In the published article, this work was not included in the list of references. The reference is “Mändle, T., Einsele, H., Schaller, M., Neumann, D., Vogel, W., Autenrieth, I. B., et al. (2005). Infection of human CD34+ progenitor cells with *Bartonella henselae* results in intraerythrocytic presence of *B. henselae*. *Blood* 106, 1215–1222. doi: 10.1182/blood-2004-12-4670”.

In the published article, there was an error in the section **Infection of endothelial cells (ECs)**, paragraph 1. The citation in “The degradation of extracellular matrix proteins has been confirmed to be facilitated by the interaction between fibrinolysis and several pathogen proteins (Vaca et al., 2022).” was incorrect. The correct citation is “(Lähteenmäki et al., 2001)” In the published article, this work was not included in the list of references. The reference is “Lähteenmäki, K., Kuusela, P., Korhonen, T. K. (2001). Bacterial plasminogen activators and receptors. *FEMS Microbiol. Rev*. 25, 531–552. doi: 10.1111/j.1574-6976.2001.tb00590.x”.

In the published article, there was an error in the section **Infection of endothelial cells (ECs)**, paragraph 2. The citation in “These adhesins belong to the trimeric autotransporter adhesin (TAA) family (Hoiczyk et al., 2000).” was incorrect. The correct citation is “(Linke et al., 2006)”. As the work is no longer cited, the reference “Hoiczyk E, Roggenkamp A, Reichenbecher M, Lupas A, Heesemann J (2000). Structure and sequence analysis of Yersinia YadA and Moraxella UspAs reveal a novel class of adhesins. *EMBO J*. 19, 5989–5899. doi: 10.1093/emboj/19.22.5989” has been removed.

In the published article, there was an error in the section **Infection of endothelial cells (ECs)**, paragraph 3. The citation in “It activates hypoxia-inducible factor-1 and stimulates the secretion of pro-angiogenic cytokines, such as vascular endothelial growth factor (VEGF) and C-X-C motif chemokine ligand (CXCL) 8 (Kaiser et al., 2008, 2012), contributing to *Bartonella*-induced vasoproliferation.” was incorrect. The correct citation is “(Riess et al., 2004; Kempf et al., 2005; McCord et al., 2006)” In the published article, the following works were not included in the list of references, but have been added:

“Riess, T., Andersson, S. G., Lupas, A., Schaller, M., Schäfer, A., Kyme, P., et al. (2004). *Bartonella* adhesin A mediates a proangiogenic host cell response. *J. Exp. Med*. 200, 1267–1278. doi: 10.1084/jem.20040500”

“Kempf, V. A., Lebiedziejewski, M., Alitalo, K., Wälzlein, J. H., Ehehalt, U., Fiebig, J., et al. (2005). Activation of hypoxia-inducible factor-1 in bacillary angiomatosis: evidence for a role of hypoxia-inducible factor-1 in bacterial infections. *Circulation* 111, 1054–1062. doi: 10.1161/01.CIR.0000155608.07691.B7”

As it is no longer cited, the reference “(1) Kaiser, P. O., Linke, D., Schwarz, H., Leo, J. C., and Kempf, V. A. (2012). Analysis of the BadA stalk from Bartonella henselae reveals domain-specific and domain-overlapping functions in the host cell infection process. *Cell. Microbiol*. 14, 198–209. doi: 10.1111/j.1462-5822.2011.01711.x” has been removed.

In the published article, there was an error in the section **Blood-sucking arthropods as vectors for** ***Bartonella*** **transmission**, paragraph 1. The citation in “… human body lice *Pediculus humanus corporis* for *B. quintana* (Kloch et al., 2018)…” was incorrect. The correct citation is “(Byam and Lloyd, 1920)”. In the published article, this work was not included in the list of references. The reference is “Byam, W., and Lloyd, L. (1920). Trench fever: its epidemiology and endemiology. *Proc. R. Soc. Med*. 13, 1–27.” As it is no longer cited, the reference “Kloch, A., Wenzel, M. A., Laetsch, D. R., Michalski, O., Bajer, A., Behnke, J. M., et al. (2018). Signatures of balancing selection in toll-like receptor (TLRs) genes - novel insights from a free-living rodent. *Sci. Rep*. 8:8361. doi: 10.1038/s41598-018-26672-2” has been removed.

The authors apologize for these errors and state that they do not change the scientific conclusions of the article in any way. The original article has been updated.
